# Reflex Nervous Disorders from Dental Irritation

**Published:** 1876-02

**Authors:** Alfred L. Carroll

**Affiliations:** University, N. Y.


### REFLEX NERVOUS DISORDERS FROM DENTAL IRRITATION.

BY ALFRED L. CARROLL, M. D., UNIVERSITY, N. Y.

*Read before the American Academy of Dental Surgery, October, 19th, 1875.*

The intimate association between dental irritation, even under conditions that can scarcely be called morbid, and disturbances of other organs is familiar to every one who has had the care of infancy and childhood. Granting that some of the ailments popularly attributed to "teething" are coincidents rather than consequences of dentition, there remains an important list of pathological phenomena unquestionably excited by reflex action from such local irritation. Although the simultaneous development of the digestive apparatus during the latter part of the first year of life may account for a predisposition to intestinal derangements, diarrhœal symptoms, not traceable to errors of diet or other violations of hygienic rules, so constantly attend the eruption of each of the first teeth, that most writers regard the bowels as a sort of safety valve to preserve the nervous system from a more disastrous explosion. Certain it is that in children who do not have this *quasi* compensatory diarrhœa we are more apt to meet with convulsions, evidently dependent on dental evolution, and especially when the sources of peripheral irritation are multiplied by the cutting of several teeth at once, do we find spasms resulting, sometimes even of tonic character. Almost all authorities are in accord as to the influence of dentition in producing laryngismus stridulus; some are inclined to consider it as an exciting factor in acute meningitis and in infantile paralysis. Every ear is conversant with the bronchial cough which in many children recurs with the appearance of each new tooth, and every eye with a host of minor nervous disturbances in teething infants.

So, too, the second dentition often brings before us evidences, more or less marked, of reflex disorders. Diarrhœal troubles, irritative cough, neuralgias of various kinds, nervous depression and nutritive disturbances, are not uncommonly associated with the eruption of the permanent canines and molars, and the frequency of chorea during this period of life seems to point to dental irritation as one of the provocatives of this latter malady. The cutaneous affections, so often connected in their outbreaks or exacerbations with the successive stages of dentition, are now generally assigned to what I believe to be their proper place among the neuroses. At a still later age, most of us have met with more or less serious reflex derangements resulting from the pressure of a developing wisdom tooth.

If such be the case even where there is but little departure from the prescribed processes of nature, we might, *a priori*, expect to find more marked results from morbid conditions affecting the dental nerves, and this expectation is fully verified by experience. The commonest consequences are neuralgias involving one or more of the divisions of the fifth pair of nerves. Toothache itself is not a necessary concomitant of caries, but often, if not always, a true neuralgia, of which the dental lesion is merely the excitant; we frequently see teeth which have decayed to the very roots, or died from other causes, without ever producing toothache, whilst, on the other hand, even oftener, from irritation or inflammation of a single tooth, both the superior and inferior dental nerves will be so generally affected that the patient can not tell whether an incisor or a molar is the offending member, or whether the real seat of pain is in the upper or under jaw.* With the various lesions besides caries which may light up and maintain persistent and agonising neuralgia, the Fellows of this

*In testimony of the neuralgic nature of toothache, Althaus adduces the fact that the suffering manifestly arising from a carious tooth may be relieved by the application of the anode of the galvanic battery, and I may add that in my own practice I have several times seen the anti-neuralgic virtue of tincture of gelseminum signally shown in the cure of pain that was located in a single tooth.

Academy are more familiar than I, and better know the parts played by exostoses, hypertrophy of the cementum and other pathological states not easily recognized by the inexpert. But the most noteworthy fact in this connection is that a neuralgia of dental origin may almost or altogether spare the dental nerves and localise itself in other nervous branches. I have repeatedly traced an attack of supra-orbital or molar neuralgia to a tooth which was sensitive to tapping, but which had given no indications nearer home of its irritating influence; and it is no uncommon thing to find cases of "tic douloureux" unmistakably arising from a bad tooth, wherein the dental nerves bear a subordinate share of the suffering. Moreover, the mischief is not confined to the branches of the trigeminus, but may pass over these to seize upon more distant nerves, the brachial and cervical plexuses not infrequently bearing the brunt of pain which is occasionally referred exclusively to one or more of their branches. In a curious case, recorded by Mr. Salter, of London, a lady was affected with neuralgia, which, beginning in the trigeminus, ultimately involved nearly all the sensory nerves of the body, and was not permanently relieved until after the extraction, at intervals, of all her teeth, every one of which, though free from caries, had its fang encroached with nodular exostoses. A somewhat similar case is described in Fox's treatise on the diseases of the teeth, wherein impairment of sight and closure of the lids of one eye, were among the symptoms caused by equally general dental exostosis, and numerous other illustrative examples abound in dental literature.

Motor and trophic disturbances, too, may be occasioned. Tomes cites a case of fatal tetanus caused by pivoting an artificial crown upon the fang of a broken tooth, and not a few instances of trismus from diseased or impacted teeth have been reported. Wryneck has been traced to the same origin and cured by extracting the offending teeth, and epilepsy has acknowledged a similar cause and yielded to similar treatment. Facial palsy and motor paralysis of the arm from this source have several times been noted, as well as temporary deafness and loss or impairment of sight, promptly relieved by the dentist's forceps. The list may seem a long one, but it might be much further extended, including various grades of nutritive disturbances, from local trophic lesions up to general malnutrition almost simulating phthisical decline. In fact, any of the reflex phenomena—and their name is legion —which are recorded as occasionally dependent upon peripheral irritation elsewhere, may take their rise from morbid dental excitation, and I believe that closer inquiry will increase the frequency with which this connection is recognized. I do not, of course, mean to imply that diseased teeth will always produce reflex derangements, any more than that a punctured wound will always cause tetanus, or a grain of sand under a toe nail necessarily excite epilepsy; but, given the exceptional neurotic temperament which is predisposed to reflex disorders, we shall probably find the starting point of these latter oftener in dental irritation than in any other peripheral lesion.

The practical outcome of the facts which I have hurriedly reviewed, is the question—which I propound with all deference to the Academy:—Is not conservative dentistry sometimes carried too far? Whilst fully appreciating the advance of dental science and gratefully admiring the skill of the modern dental surgeon in saving many a useful tooth which would have been sacrificed by his predecessors, it seems to me that there is danger of this skill being concentrated too exclusively upon the special organ in hand, overlooking graver remote complications. I have noticed for many years past a growing reluctance to use the forceps—a most desirable reaction, I grant, from the barbarous days when every tooth that ached was remorselessly pulled, and every schoolboy's holidays were haunted with inquisitorial nightmares of turnkeys and like instruments of torture; but with the possible drawback that what is a brilliant local success from the specialist's point of view, may maintain and aggravate evils far greater than the loss of a tooth. Prof. Cartwright, of King's College, London, goes even so far as to predict that "the day is at hand when the necessity of stopping or extracting a tooth will be looked upon, to a certain extent, as a confession of failure in treatment,"* and I believe that he is not singular in his hopeful estimate of dental therapeutics. This view, however, overlooks the fact that a tooth may be virtually as much a foreign body as a bullet or a splinter, coming under the general rule of surgery which enjoins the removal of any mechanical source of irritation; and that, moreover, the idiosyncrasy of one patient may demand the sacrifice of a tooth which might be saved if it were in another person's head. In the case of an unimpressible nervous system, where neuralgic pain, if it be present at all, is confined to the branches immediately irritated and manifestly due to a temporary accident, cosmetic considerations may prevail and an exposed pulp may be bridged over, or an alveolar abscess treated through the fang-cavity or any of the admirable conservative exploits of modern dentistry accomplished with perfect propriety; but where we have to deal with a neurotic temperament, with neuralgias or other nervous manifestations more persistent in duration and reflected to remoter branches, the aspect of affairs is altogether changed and in such instances any attempt to treat the dental lesion *in situ* will be too apt to prolong and increase the patient's sufferings. For example; I was not long ago consulted by a lady who had for some months been afflicted with facial neuralgia chiefly affecting the supra-orbital and malar branches. Although there had been no complaint of distinct toothache, I found an upper bicuspid on the affected side very sensitive on tapping but presenting to my untutored eye no external signs of disease, and, regarding this as the probable cause of the trouble, I advised the patient to have it removed. A day or two afterwards I saw her after a sleepless night, with all her pains much aggravated and their range extended, and discovered that an india-rubber wedge had been inserted between the tender tooth and its neighhor, augmenting the local irritation tenfold and unpleasantly substantiating my diagnosis. I can readily understand how the dental surgeon may be loth to create a gap in a pretty woman's mouth—I doubt if I should have the courage to do it myself—but in

*Brit. Med. Jour., July 17, 1875.

the instance in question (which is but a sample of many similar ones) it would indisputably have been to the patient's advantage to have had recourse at first to Colton's inartistic gas. So, too, I have seen teeth carefully and beautifully filled where subsequent extraction, necessitated by persistent neuralgia, showed exostosis of their fangs or nodules encroaching on their cavities. And I think I have seen more than one case where the mechanical force required for a gold filling in a tooth whose life was, so to speak, hanging by a thread, has turned the balance to irreparable inflammation and lighted up a train of reflex disturbances which might not otherwise have occurred. For this reason I believe that one of the greatest desiderata of future dental surgery is some durable substance which may be moulded without violence to a cavity in a tender tooth; and until this want shall be satisfactorily supplied I am an earnest advocate to gutta-percha where it can be made available in such cases, and for grinding surfaces, should prefer the much abused amalgam to gold. I know the argument often advanced, that a tooth which can bear the power employed in mastication ought to be able to bear the force employed in packing a gold filling in it; but it must be considered, first, that the power of the temporal, masseter and pterygoid muscles is at all times distributed over a larger surface; secondly, that a tender tooth is instinctively spared from taking even its ordinary share in the process of chewing; and thirdly, that the yielding character of the substances used as food renders the gradual impact of the teeth a different thing from the inelastic action of the plugging instrument—still more different from the sudden jar of the mallet. It is a matter of daily observation with all of us that a tooth which the patient has used in eating without inconvenience, is found to be exceedingly sensitive to a gentle tap from any hard body and it seems to stand to reason that in such a case repeated shocks—especially if applied laterally—may easily aggravate the existing mischief, and, in a fitting subject, cause its extension to a wider range of the nervous apparatus.

I am travelling beyond my sphere, perhaps, in presuming to enter upon the practical details of dental surgery, but my experience of neuralgias which, on tracing back their history, appeared to be connected with some well remembered painful filling, convinces me that the question to which I have just adverted, deserves to be submitted to the judgment of the Academy; and its difficulty is enhanced by the circumstance, which we may infer from analogous peripheral irritations in other situations, that the irritant may maintain its remoter reflex consequences long after the immediate seat of its action has become habituated to its presence and ceased to exhibit any local manifestations of its influence; in other words, that symptoms elsewhere may be sustained by a tooth whose tenderness has been allayed by time, leaving no consciousness of trouble in the vicinity of its alveolus. We have seen that toothache is not a necessary preliminary or accompaniment of disturbances indisputably reflected from the dental branches of the fifth nerve; nay, we know that the mere overcrowding of perfectly sound teeth will occasionally originate such disturbances in susceptible persons; and, *a fortiori*, I think it may be assumed that a tooth which has given evidence of its incendiary tendency is open to suspicion, even after its seeming reformation, when we find burning in its nervous district, not otherwise to be accounted for.

Neither your time nor my opportunity has permitted me to do more than offer a few suggestions which may be more wisely elaborated in the discussions of the Academy; but I trust I have said enough to show that the pathological territory upon which dental surgery and pure medicine meet, is wider than is commonly imagined and I would urge upon the young physician, in the words of a most observant medical writer,* that:—

"The importance of investigating the state of the mouth in all cases of neuralgia about the face and head, headache, deafness, amaurosis, facial paralysis, epilepsy, abscess of the antrum and hypochondriasis, can not be too strongly insisted upon;" whilst to the young dental surgeon I would commend the propriety of recognizing the nervous system as an appen-

*Tanner, Practice of Medicine.

dage to the teeth and bearing in mind the reflex disorders which; in certain temperaments, his very skill, too specially applied, may possibly perpetuate.

